# *PUCHI* Regulates Giant Cell Morphology During Root-Knot Nematode Infection in *Arabidopsis thaliana*

**DOI:** 10.3389/fpls.2021.755610

**Published:** 2021-10-06

**Authors:** Reira Suzuki, Mizuki Yamada, Takumi Higaki, Mitsuhiro Aida, Minoru Kubo, Allen Yi-Lun Tsai, Shinichiro Sawa

**Affiliations:** ^1^Department of Biological Sciences, Graduate School of Science and Technology, Kumamoto University, Kumamoto, Japan; ^2^International Research Organization for Advanced Science and Technology, Kumamoto University, Kumamoto, Japan; ^3^International Research Center for Agricultural and Environmental Biology, Kumamoto University, Kumamoto, Japan; ^4^Center for Digital Green-innovation, Nara Institute of Science and Technology, Nara, Japan

**Keywords:** plant-parasitic nematode, cell wall, cell morphology, VLCFA, plant-pathogen interaction

## Abstract

Parasitic root-knot nematodes transform the host’s vascular cells into permanent feeding giant cells (GCs) to withdraw nutrients from the host plants. GCs are multinucleated metabolically active cells with distinctive cell wall structures; however, the genetic regulation of GC formation is largely unknown. In this study, the functions of the *Arabidopsis thaliana* transcription factor PUCHI during GC development were investigated. *PUCHI* expression was shown to be induced in early developing galls, suggesting the importance of the *PUCHI* gene in gall formation. Despite the *puchi* mutant not differing significantly from the wild type in nematode invasion and reproduction rates, *puchi* GC cell walls appeared to be thicker and lobate when compared to the wild type, while the cell membrane sometimes formed invaginations. In three-dimensional (3D) reconstructions of *puchi* GCs, they appeared to be more irregularly shaped than those in the wild type, with noticeable cell-surface protrusions and folds. Interestingly, the loss-of-function mutant of *3-KETOACYL-COA SYNTHASE 1* showed GC morphology and cell wall defects similar to those of the *puchi* mutant, suggesting that *PUCHI* may regulate GC development *via* very long chain fatty acid synthesis.

## Introduction

Plant-parasitic nematodes, such as root-knot nematodes (RKNs, *Meloidogyne* spp.), are known to infect and damage a broad spectrum of crops. RKNs feed on the vascular cells of host plant roots, which impedes plant development and may even lead to plant death. Infective RKN second instar (J2) larvae enter the host plant roots and migrate to the vasculature, where they induce the formation of special feeding sites called galls. It is thought that RKNs inject various effector proteins into vascular cells, which convert these cells into specialized feeding cells known as giant cells (GCs; Williamson and Gleason, 2003).

Galls play an essential role in sustaining RKN development (Favery et al., 2016), and GCs form the majority of a gall’s volume and provide RKNs with nutrients. The development of GCs thus directly dictates gall formation and RKN development. GCs result from repeated nuclear divisions without cell division (endoduplication) and isotropic plant cell growth (Caillaud et al., 2008). The first sign that RKNs are manipulating cell development is the appearance of supernumerary nuclei. Subsequently, cell plate formation is inhibited, and these multinucleated cells continue to endoduplicate until they contain up to 100 nuclei (Wiggers et al., 1990). GCs also contain cytoplasm densely packed with organelles and often show a high level of metabolic activity; thus, they may be up to 400 times larger than normal vascular cells (Caillaud et al., 2008). Nutrient uptake into GCs from the vasculature is enhanced by the development of numerous cell wall ingrowths adjacent to the xylem contact site (Jones and Payne, 1978; Bartlem et al., 2014). Furthermore, the constant withdrawal of nutrients by RKNs converts GCs into metabolic sinks for host plants (Favery et al., 2016). However, the molecular mechanisms underlying GC formation mediated by RKNs largely remain to be elucidated.

Galls can be considered a novel organ formed from root tissues during RKN infection, with development mechanisms similar to those of lateral roots (LRs) and adventitious roots. Recent studies have suggested that the developmental processes of RKN-induced galls, LRs, and calluses have overlapping regulatory mechanisms. Hence, genes that are known to be upregulated in LRs and calluses, such as master regulatory transcription factors, have also been identified in galls (Cabrera et al., 2014; Olmo et al., 2017, 2020). In addition, plant-parasitic nematodes are known to modulate the hosts’ vascular development during gall formation (Yamaguchi et al., 2017). Currently, it remains unclear how LR development regulatory pathways are adapted to control GC development.

*PUCHI* encodes an APETALA2/ethylene-responsive element-binding transcription factor found exclusively in plants that regulates the differentiation of lateral organs in shoots and roots (Hirota et al., 2007; Karim et al., 2009). In shoots, *PUCHI* is involved in the determination of floral meristem identity and the suppression of bract growth (Karim et al., 2009), while in roots, *PUCHI* restricts stem cell proliferation and coordinates cell division patterns during lateral root formation (Hirota et al., 2007). Moreover, *PUCHI* has been shown to be induced during LR initiation (Goh et al., 2019), and recent studies have revealed that *PUCHI* controls LR development, at least in part, through regulation of very long chain fatty acid (VLCFA) biosynthesis (Goh et al., 2019; Trinh et al., 2019). VLCFAs have 20 or more carbon atoms in the backbone, are synthesized by the fatty acid elongase complex, and are components of various membrane, storage, and extracellular lipids (Li-Beisson et al., 2013). *PUCHI* activates a subset of VLCFA biosynthetic genes to regulate cell proliferation during LR development (Trinh et al., 2019; Guyomarc’h et al., 2021). Essentially, *PUCHI* ensures proper organogenesis through transcriptional regulation, while repressing the development of other tissues during flowering and LR development. In addition, *PUCHI* has been shown to function during callus formation (Trinh et al., 2019). However, the role of *PUCHI* during gall formation remains unclear. The results of an RNA-Seq analysis of a series of developing galls suggested that *PUCHI* is upregulated during gall formation (Yamaguchi et al., 2017). Here, we further examine the functions of *PUCHI* during RKN infection.

## Materials and Methods

### Plant Materials and Growth Conditions

*Arabidopsis thaliana* of the Columbia-0 (Col-0) ecotype, which includes *puchi-1*, *puchi-2* (Henikoff et al., 2004; Hirota et al., 2007), *kcs1-5* (SALK_200839; Shang et al., 2016), and *pKCS1::GUS* (Joubès et al., 2008), was used for this study. To construct *ProPUCHI-GUS-tPUCHI*, the *PUCHI* promoter (−1 to −3,892 bases upstream of the start codon) and *PUCHI* terminator (+1 to +1,743 bases downstream of the stop codon) sequences were cloned upstream and downstream of the beta-glucuronidase (GUS) gene, respectively. The expression cassette was then cloned into the binary vector pBIN50 (Takano et al., 2010). The construct was used to transform Col-0 and *puchi-1* plants. Seeds were surface-sterilized and sown on plates with Murashige and Skoog (MS) media [0.25× MS salt mixture (Sigma-Aldrich), 0.5% (w/v) sucrose, and 0.6% (w/v) phytagel or gellan gum, pH 6.4], vernalized for 2days in the dark at 4°C, then incubated and grown under continuous light at 23°C.

### Nematode Preparation and Inoculation

Root-knot nematodes were propagated as previously described (Nishiyama et al., 2015). Briefly, 6- to 7-week-old tomato (*Solanum lycopersicum*) cultivar Pritz plants were inoculated with 20,000 RKN J2 larvae at 3-day intervals for a total of four inoculations (80,000 J2s). The inoculated tomato plants were then transferred into hydroponics systems (Nishiyama et al., 2015), and newly hatched J2s were harvested at 2- to 4-day intervals from the hydroponic culture media and surface-sterilized before use.

For RKN-infection assays, six 5-day-old *Arabidopsis* seedlings were grown in a petri dish with MS media, inoculated with approximately 80 sterilized J2s per plant, and incubated under short-day conditions (8h light and 16h dark) at 25°C. The roots of the seedlings were covered with black paper to mimic nature’s dark underground environment. Galls, adult females, and egg masses were counted at 14, 28, and 42days post-infection (dpi), respectively.

### Histological Analyses

For acid fuchsin staining, the roots of RKN-infected seedlings were washed in deionized water and then transferred into 1% (v/v) sodium hypochlorite for 10–30min. Roots were then washed twice with deionized water and boiled in a solution of 30-fold diluted acid fuchsin stock [25% (v/v) acetic acid and 0.035% (w/v) acid fuchsin] for 10min. Cooled samples were washed with deionized water twice and then incubated in acidified glycerol (1.2-mm hydrochloric acid in glycerol) for 2–15min at 95°C. Samples were then mounted in acidified glycerol and observed using an Axio Imager M1 microscope (Carl Zeiss) equipped with a DP71 digital camera (Olympus).

For GUS staining, roots were fixed in 90% acetone for 24h at −30°C and stained in GUS buffer [100-mm NaPO_4_ buffer (pH 7), 10-mm ethylenediaminetetraacetic acid (EDTA, pH 8), 0.1% Triton X-100, 3-mm K_3_Fe(CN)_6_, and 3-mm K_4_Fe(CN)_6_] with 0.5mg/ml 5-bromo-4-chloro-3-indolyl-beta-D-glucuronide (Wako) for 4h. The reaction was stopped with Carnoy’s solution [90% (v/v) methanol and 10% (v/v) acetic acid], and the roots were mounted in chloral hydrate solution [80%(w/v) chloral hydrate and 10% (v/v)glycerol]. Samples were observed using an Axio Imager M1 microscope (Carl Zeiss Microscopy) and photographed using a DP71 digital camera (Olympus).

For toluidine blue-stained sections, galls were dissected and transferred into 2% (v/v) glutaraldehyde in 20-mm cacodylate buffer (pH 7.4), vacuum-infiltrated twice for 10min each, and then incubated at 4°C overnight. Samples were dehydrated in a graded ethanol series (50% ethanol for 10min, 75% ethanol for 10min, 90% ethanol for 10min, 95% ethanol for 10min at room temperature, and anhydrous ethanol for 20min at room temperature) and embedded in Technovit 7100 (Kulzer) according to the manufacturer’s instructions. Sample blocks were then sectioned to 5μm thickness using an ultramicrotome (Leica RM2255) and stained with 0.01% (w/v) toluidine blue O (WALDECK) containing 1% (w/v) sodium borate decahydrate (Nacalai) for 2min. Samples were then rinsed in deionized water for 1min, dried, and mounted in EUKITT (O. Kindler). Samples were observed using an Axio Imager M1 microscope (Carl Zeiss) equipped with a DP71 digital camera (Olympus).

### Gene Expression Analysis

Total RNA was extracted from either 40 roots or 50 galls using the RNeasy plant mini kit (Qiagen) with the RNase-Free DNase Set (Qiagen) according to the manufacturer’s instructions. First-strand cDNA was synthesized from 300ng of total RNA using the PrimeScript RT Master Mix (Takara). Transcript levels were assayed using the FastStart Essential DNA Green Master (Roche) and the LightCycler 480 system (Roche). The thermal cycler program consisted of 95°C for 5min followed by 55cycles of 95°C for 10s, 60°C for 10s, and 72°C for 10s. The glyceraldehyde 3-phosphate dehydrogenase (*GAPDH*) gene was used as the internal control to calculate relative expression levels. Each of the three biological replicates was performed in technical triplicate. Real-time polymerase chain-reaction (RT-PCR) analyses were performed with the specific primers (see Supplementary Table 1). In all experiments, expression levels were normalized against those of *GAPDH*.

### Microscopy

For transmission electron microscopy (TEM) analyses, samples were fixed in 2% paraformaldehyde (w/v) and 2% glutaraldehyde (v/v) in 0.05-M cacodylate buffer (pH 7.4) at room temperature and then refrigerated at 4°C. Samples were then fixed in 2% glutaraldehyde in 0.05-M cacodylate buffer (pH 7.4) at 4°C overnight. After fixation, the samples were washed three times with 0.05-M cacodylate buffer for 30min each and post-fixed with 2% osmium tetroxide (w/v) in 0.05-M cacodylate buffer at 4°C for 3h. The samples were dehydrated in graded ethanol solutions (50% ethanol for 30min at 4°C, 70% ethanol for 30min at 4°C, 90% ethanol for 30min at room temperature, and three changes of anhydrous ethanol for 30min each at room temperature). Samples were further dehydrated in anhydrous ethanol at room temperature overnight. Then, the samples were infiltrated with propylene oxide twice for 30min each, placed into a 70:30 mixture of propylene oxide and resin (Quetol-651; Nisshin EM) for 1h, and then incubated overnight with the cap open to evaporate the excess propylene oxide. The samples were transferred to fresh 100% resin and polymerized at 60°C for 48h. The resin-embedded samples were first sectioned to 1.5μm with a glass knife using an ultramicrotome (Ultracut UCT; Leica, Vienna, Austria) and stained with 0.5% toluidine blue. Ultra-thin sections of 80nm were then prepared using a diamond knife and an ultramicrotome (Ultracut UCT; Leica, Vienna, Austria) and mounted on copper grids. The sections were stained with 2% uranyl acetate at room temperature for 15min, washed with distilled water, and stained with lead stain solution (Sigma-Aldrich) at room temperature for 3min. The grids were observed under TEM (JEM-1400Plus; JEOL Ltd., Tokyo, Japan) at an acceleration voltage of 100kV. Images were taken with a charge-coupled device camera (EM-14830RUBY2; JEOL Ltd.)

Samples imaged by confocal microscopy were first cleared with ClearSee [10% (w/v) xylitol, 15% (w/v) sodium deoxycholate, and 25% (w/v) urea] (Kurihara et al., 2015). Galls were vacuum-infiltrated twice with 4% (w/v) paraformaldehyde for 30min each at room temperature. Fixed tissues were cleared with ClearSee at room temperature for 1week or longer, depending on tissue type (Kurihara et al., 2015). For post-staining, cleared tissues were stained with Calcofluor White (100μg/ml) and 1/1,000 SYBR Green 1 in ClearSee for 1h. After staining, tissues were washed in ClearSee for 2min. Samples were imaged with a confocal laser microscope (FV3000, Olympus) with an Olympus FV 10-MCPSU (405nm and 488nm) and ×60, NA 1.35 oil objective (UPlanSApo, Olympus). All Z-stack sections were imaged at 1–1.5-μm intervals.

### Image Analyses

A pretrained deep learning model based on a convolutional neural network in AIVIA (DRVision, Bellevue, WA, United States) was used to identify GCs from confocal images. A total of 17 binary images in which the cell regions were manually segmented were used as the training data set. The output images were binarized with manual intensity thresholding to determine the cell regions. The surface models for the 3D cell reconstruction were obtained using Imaris (Bitplane, Belfast, UK).

## Results

### *PUCHI* Is Expressed in RKN Feeding Sites

**Figure 1:**
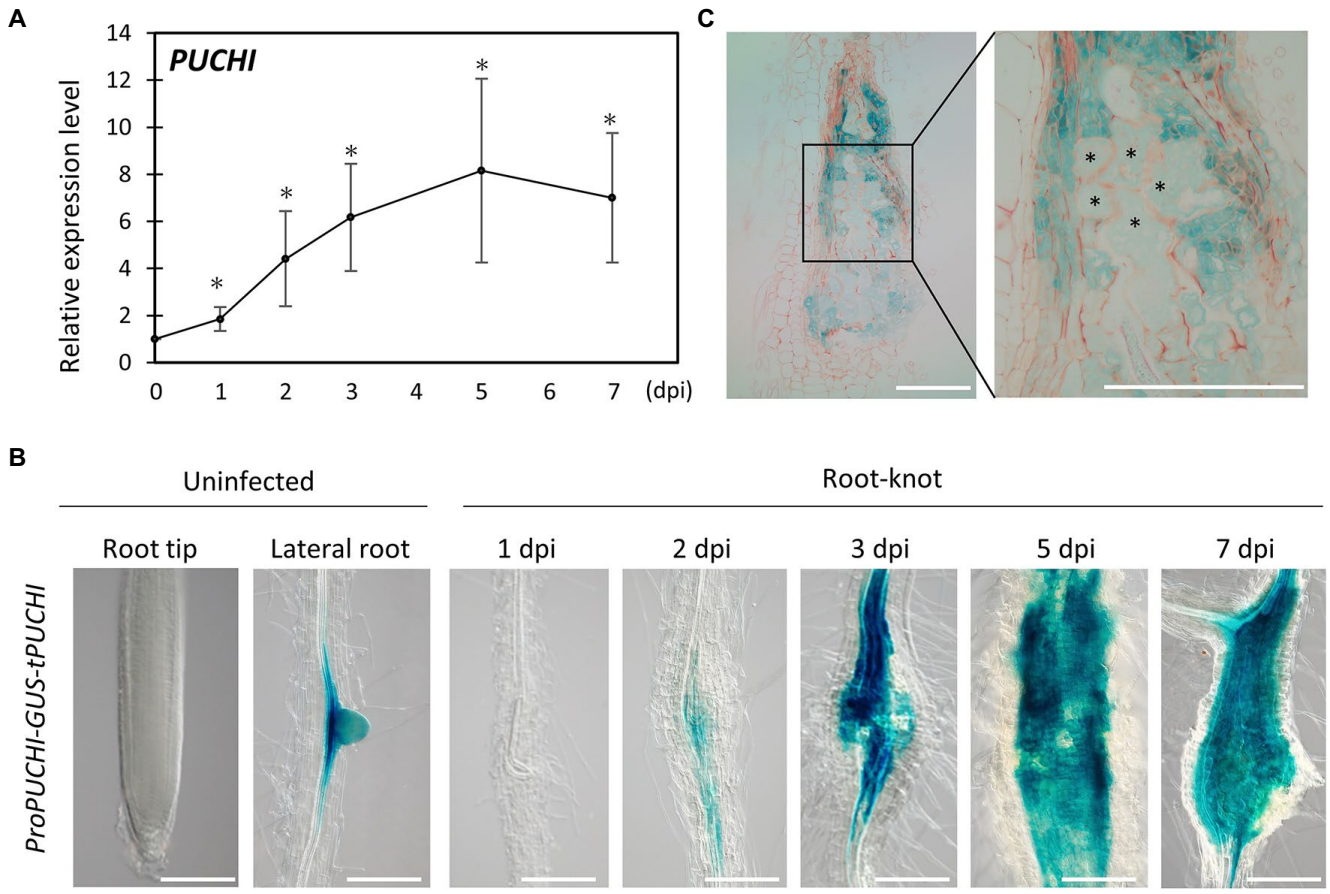
*PUCHI* expression analysis. **(A)** Quantitative RT-PCR results of *PUCHI* transcript levels in 1–7 dpi galls and uninfected roots. Means ± standard error is shown. ^*^p<0.05 using Steel’s multiple comparison test. **(B)** GUS-stained *ProPUCHI-GUS-tPUCHI* 1–7 dpi galls and uninfected roots to denote the spatial expression patterns of *PUCHI.* **(C)** GUS-stained cross-sections of 7 dpi *ProPUCHI-GUS-tPUCHI* galls. ^*^ denotes GCs. Scale bars: 100μm.

RNA-Seq data from developing galls showed that *PUCHI* was upregulated during gall formation (Supplementary Figure S1A; Yamaguchi et al., 2017). This suggests that *PUCHI* may play important roles related to RKN infection. Indeed, quantitative reverse transcription polymerase chain-reaction (qRT-PCR) results are consistent with the notion that RKN infection causes the accumulation of *PUCHI* transcripts, since *PUCHI* mRNA levels increased as gall formation progressed (Figure 1A). *PUCHI* expression peaked by 5 dpi when the transcript level increased 8-fold compared to uninfected roots (Figure 1A).

To confirm the spatial expression pattern of *PUCHI* after RKN infection in wild-type (WT) plants, transgenic plants carrying *ProPUCHI-GUS-tPUCHI* construct in the WT background were examined. GUS expression was observed mostly in the lateral root primordia (LRP) of uninfected roots, which is consistent with previous work (Figure 1B; Hirota et al., 2007). In contrast, GUS expression was seen in the swelling central cylinder in the roots at 2 dpi and became more pronounced by 7 dpi (Figure 1B). Specifically, GUS expression in galls was primarily in neighboring cells surrounding the GCs, while weaker GUS staining was detected in the GCs (Figure 1C). *PUCHI* may function by mediating signal transductions from neighboring cells to GCs and regulating GC development non-cell autonomously. Taken together, these lines of evidence indicate that *PUCHI* is specifically induced during gall formation, especially in the early stages. This in turn suggests that *PUCHI* may play a role during gall formation.

Since *PUCHI* encodes a transcription factor, it is possible that *PUCHI* regulates its own expression. To determine whether this was the case, the *ProPUCHI-GUS-tPUCHI* reporter construct was also analyzed in the *puchi-1* background. Similar to the situation in the WT background, GUS expression was also detected in the LRP and galls in *puchi-1* plants (Supplementary Figure S1B), suggesting that *PUCHI* is unlikely to regulate its own expression.

### *PUCHI* Does Not Strongly Influence RKN Infection and Development

**Figure 2:**
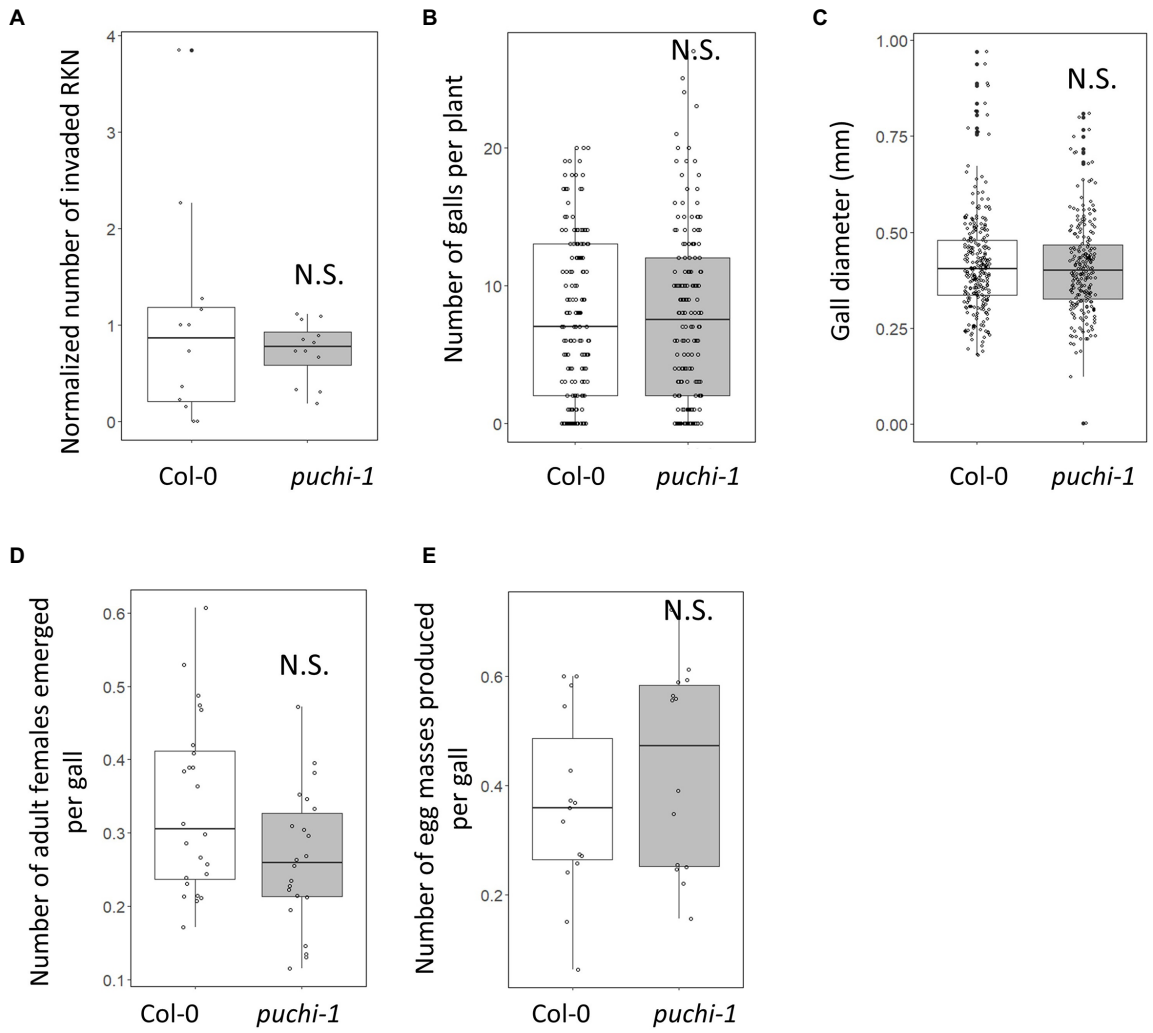
Effect of *puchi* mutation during RKN infection. Numbers of invading RKNs at 2 dpi are averages from four seedlings that were then normalized to values of WT in a root **(A)**, galls at 14 dpi **(B)**, gall diameters at 14 dpi **(C)**, emerged mature females at 28 dpi **(D)**, and egg mass at 42 dpi **(E)** in Col-0 and *puchi-1* seedlings. Values are the means of at least three biological replicates. Statistical significance was analyzed using the Brunner-Munzel test. N.S. denotes not statistically significant.

Since RKN infections induce *PUCHI* expression in feeding sites, the functions of *PUCHI* during gall formation were investigated. Infection efficiency and gall growth were assayed in the presence of *puchi-1* and *puchi-2* loss-of-function mutations. Compared to the WT, these mutations did not significantly affect RKN invasion or gall formation rates (Figures 2A,B; Supplementary Figure S2A). We also measured gall diameters in these mutants and confirmed that the average *puchi* gall sizes were comparable to that of the WT (Figure 2C; Supplementary Figure S2B). Moreover, when compared to the WT, no significant differences in the numbers of emerging adult females and egg masses were found (Figures 2D,E). These results suggest that *PUCHI* alone does not regulate RKN invasion and development rates.

### *PUCHI* Plays a Role in Maintaining GC Shape During Gall Development

**Figure 3:**
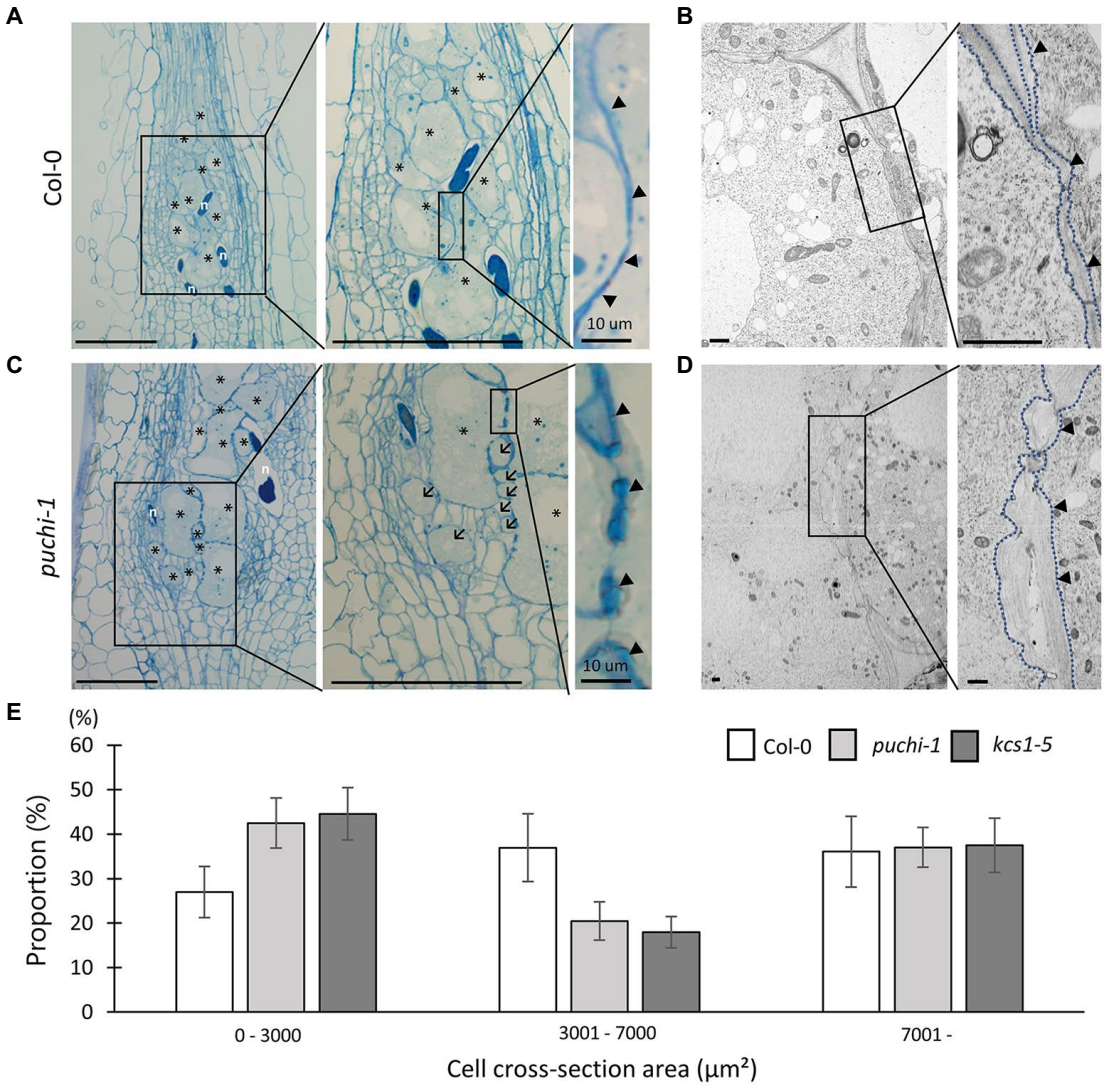
Observation of WT and *puchi-1* GC morphology. Morphological analysis of *puchi-1* and WT GCs. (A, C) Toluidine blue-stained 7 dpi WT **(A)** and *puchi-1* **(C)** gall longitudinal sections. Arrows denote aberrant GCs, and arrowheads denote cell wall thickening in Col-0 and *puchi-1* galls. ^*^ denotes GCs; n denotes RKNs. Scale bars: 100μm. (B, D) TEM micrographs of WT **(B)** and *puchi-1* **(D)** gall cross-sections. Blue dotted lines denote GC cell walls in Col-0 and *puchi-1* GCs. Arrowheads denote cell wall thickening in Col-0 and *puchi-1* GCs. Scale bars: 1μm. **(E)** Proportions of WT, *puchi-1*, and *kcs1-5* GCs with different cross-section area classes. Means ± standard deviation is shown. (WT: *n*=12, *puchi-1*: *n*=15, and *kcs1-5*: *n*=15).

To further investigate the role of *PUCHI* in developing galls, histological analyses were performed on WT and *puchi-1* galls. RKN infections cause a sophisticated transformation of root cells into GCs to nourish the RKN; this in turn results in root swellings and gall formation (Bird, 1961). Typically, 4–10 metabolically hyperactive GCs larger than other root cells were found in a single gall (Figure 3A). TEM revealed that GCs contained multiple (often endopolyploid) nuclei (Supplementary Figure S3A) and a cytoplasm densely packed with organelles (Figure 3B; Bartlem et al., 2014; Rodiuc et al., 2014).

In general, the cell walls between adjacent GCs appeared irregular and uneven in cross-sections compared to those of uninfected cells (Figure 3A right panel arrowheads; Figure 3B; Rodiuc et al., 2014). Cell wall thickness in the *puchi-1* mutant appeared more irregular than in WT, to the point where *puchi-1* GC cell walls appeared lobate in certain regions (Figure 3C right panel arrowheads; Figure 3D). Moreover, *puchi-1* cell walls often formed concave invaginations that were not found in the WT (Supplementary Figure S3C).

Interestingly, small GC-like cells could be identified in *puchi-1* 7 dpi galls, which we tentatively named “aberrant GCs” (Figure 3C center panel arrows; Supplementary Figure S3B). The *puchi-1* aberrant GCs were indeed multinucleated like normal GCs (Supplementary Figure S3B), suggesting that they were likely also products of RKN infection. To determine whether the numbers of aberrant GCs were indeed significantly higher in the *puchi-1* mutant, GC distributions (based on size) were characterized in 14 dpi galls, the time at which GC differentiation is thought to be near completion. The *puchi-1* galls showed an increase in the proportion of GCs with a cross-section area of 0–3,000μm^2^, and a decrease in those with an area of 3,001–7,000μm^2^ (Figure 3E). This result also suggests that the GC size distribution shifts toward smaller cells in the *puchi-1* mutant, confirming that the aberrant GC phenotype is quantifiable.

At later stages of gall formation (28~42 dpi), the *puchi* GC defects became more pronounced (Supplementary Figure S3D). Around 42 dpi, mature females emerge from the roots to produce egg masses. Additionally, *puchi* aberrant GCs appeared to be more abundant during late gall development (Supplementary Figure S3D).

### *Puchi* Produces GCs With Abnormal Morphology

**Figure 4:**
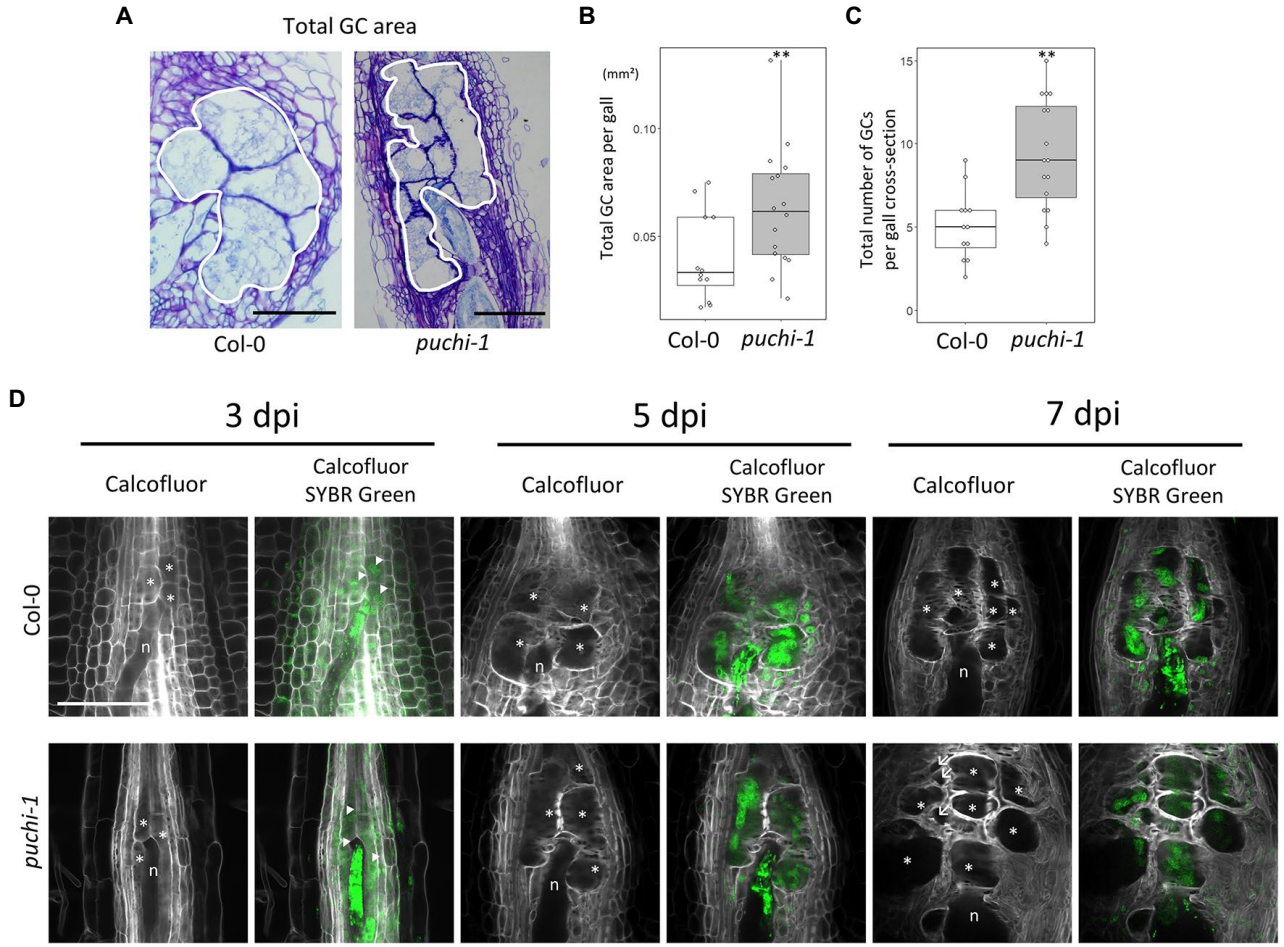
Development of WT and *puchi-1* GCs. **(A)** Areas occupied by GCs in the WT and *puchi-1* galls, as outlined with white borders. **(B)** Box plots of the total area occupied by GCs in WT and *puchi-1* galls at 14 dpi. **(C)** Box plots of the number of GCs within the area occupied by GCs in cross-sections of WT and *puchi-1* galls at 14 dpi. ^**^p<0.01, Brunner-Munzel test. **(D)** Confocal microscopy images of WT and *puchi-1* galls stained with Calcofluor White for cell walls and SYBR-Green for nuclei at 3, 5, and 7 dpi. n denotes RKNs. ^*^denotes GCs. Arrow denotes aberrant GCs. Arrowheads denote nuclei.

To further evaluate how aberrant GCs influence gall formation in *puchi* mutants, GC cross-section areas and cell numbers were assayed in both WT and *puchi* galls. Histological analysis of cross-sections through the center of galls showed that the total area occupied by GCs at 14 dpi (Figure 4A) was significantly greater in the *puchi-1* mutant than in the WT (Figure 4B). It is likely that the observed increase was due to the presence of the aberrant GCs. To address this possibility, the number of GCs per gall was counted in the WT and *puchi-1* galls. The *puchi-1* galls indeed contained significantly more GCs than the WT galls (Figure 4C).

To determine the time at which the number of GCs increased in the *puchi-1* galls, the early stages of GC development in *puchi* galls were observed using confocal microscopy (Figure 4D). SYBR Green 1 was used to stain the nuclei, and Calcofluor White was used to stain the cell wall to visualize the GCs during the early stages of gall development (Figure 4D). *puchi-1* gall cross-sections were indistinguishable from those of the WT around 3 dpi (Figure 4D). However, aberrant GCs started to appear at 7 dpi (Figure 4D). This suggests that aberrant GCs are likely to be products of defective GC formation caused by the *puchi* mutation, and not caused by factors present before RKN infection.

**Figure 5:**
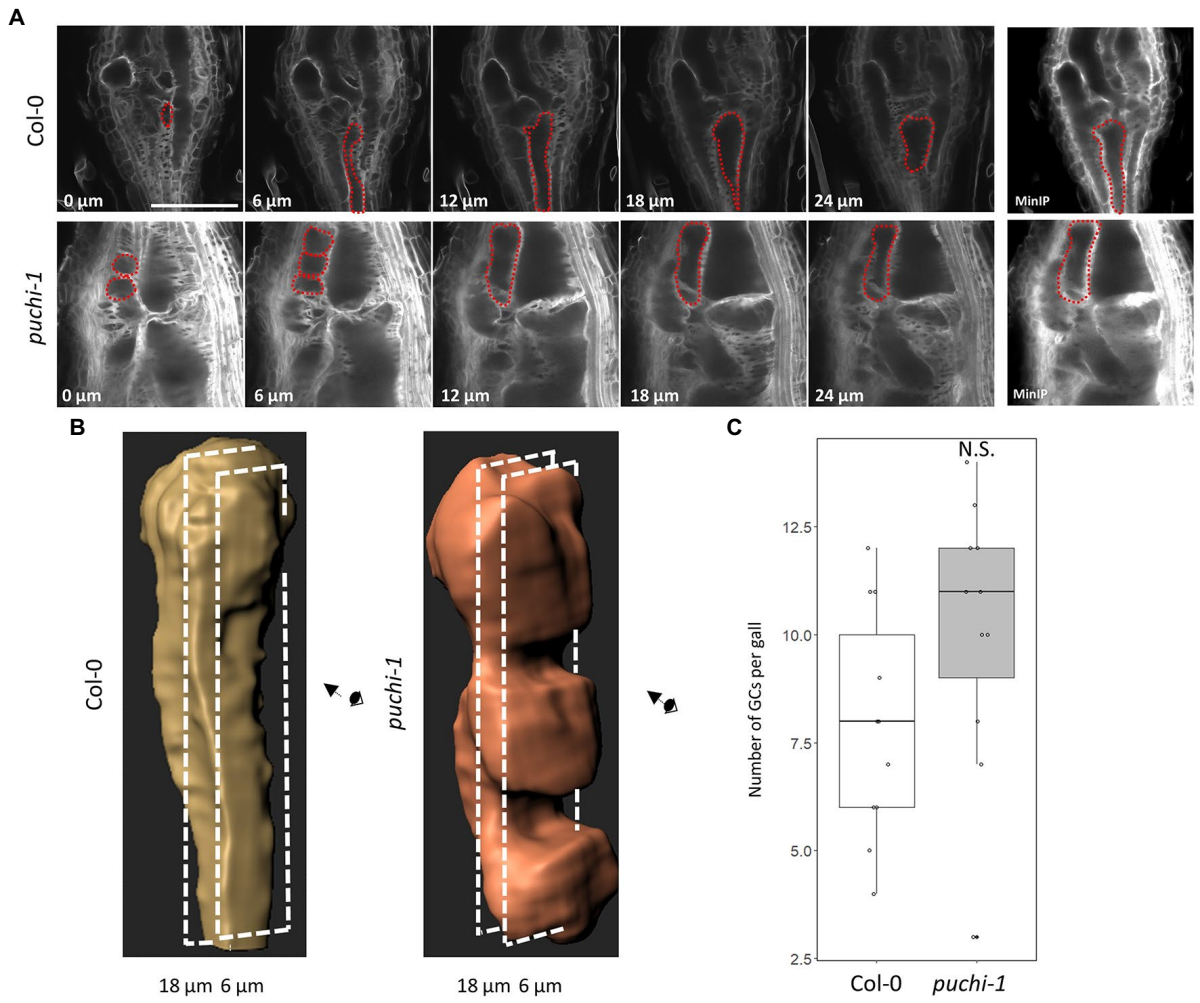
Reconstruction of 3D structures of WT and *puchi-1* GCs. **(A)** Confocal optical sections of WT and *puchi-1* galls stained with Calcofluor White at 7 dpi, and red dashed lines outline the same GC in different sections and the minimum intensity projection (MinIP) of the WT and *puchi-1* optical section Z-stacks. Scale bars: 100μm. **(B)** 3D reconstructions of individual WT and *puchi-1* GCs using the optical sections in (A). **(C)** Box plot of the number of GCs per WT and *puchi-1* gall at 7 dpi calculated using GC 3D reconstructions. Statistical significance was analyzed using the Brunner-Munzel test; N.S. denotes no statistical significance.

Next, to better characterize the 3D structure of aberrant GCs, Z-stack optical sections were used to build the minimum intensity projection (MinIP) of the WT and *puchi* GCs (Figure 5A; Supplementary Figure S4A). MinIP is used to visualize 3D structures with low intensity (Karnowski et al., 2017). Surprisingly, several *puchi* aberrant GCs that appeared as separate cells in one optical section were shown to be the same cells in different optical sections (Figure 5A; Supplementary Figure S4A). Next, the 3D structures of GCs in the WT and *puchi-1* galls were reconstructed from Z-stack optical sections using Imaris. The 3D reconstructions clearly showed that the *puchi-1* aberrant GCs were protrusions of larger GCs in other optical sections (Figure 5B; Supplementary Figure S4B). Similar results were obtained from the toluidine blue-stained sections (Supplementary Figure S4C). In WT galls, GCs did not appear to have major concavities and convexities on the surface (Figure 5B; Supplementary Figure S4B). In contrast, the *puchi-1* GCs contained dramatic protrusions and invaginations (Figure 5B; Supplementary Figure S4B). When the total number of GCs was counted using 3D reconstructions instead of cross-sections, there was no significant difference between the WT and *puchi-1* numbers (Figure 5C). These results suggest that although *puchi* does produce aberrant GCs, *puchi* galls do not contain more GCs. Rather, *puchi* produces normal numbers of normal-sized GCs with aberrant protrusions and invaginations, which may appear in single optical sections as multiple small GC-like cells.

### *PUCHI* May Regulate Gall Formation *via* VLCFA Biosynthesis

A recent study revealed that *PUCHI* controls LR development, at least in part, through the regulation of VLCFA biosynthesis (Trinh et al., 2019). VLCFAs are synthesized by the VLCFA elongase complex through a four-step process: condensation by beta-ketoacyl-CoA synthase, reduction by beta-ketoacyl-CoA reductase, dehydration by hydroxyacyl-CoA dehydratase, and reduction by enoyl-CoA reductase (Li-Beisson et al., 2013). Interestingly, the mutants with defects in the VLCFA hydroxyacyl-CoA dehydratase *PASTICCINO2* (*PAS2*) showed cell plate vesicles tethered to the plasma membrane, which superficially resembles the lobate GC cell wall found in *puchi* mutants (Bach et al., 2011; Figure 3D). It has been suggested that membrane lipids may be an important factor in mediating *PUCHI*’s role in gall formation. Therefore, we investigated whether *PUCHI* regulates the expression of VLCFA biosynthesis genes during gall formation. According to RNA-Seq data generated using developing galls (Yamaguchi et al., 2017), the expression levels of the major VLCFA elongation key enzymes fluctuated, albeit only mildly, over the course of gall formation (Supplementary Figure S5A). This suggested that these genes may be transcriptionally regulated over the course of gall formation. In galls, it was found that *3-KETOACYL-COA SYNTHASE 1* (*KCS1*) and *KCS20* were among the most highly expressed VLCFA biosynthesis genes during gall formation and that the expression peaked at around 5dpi similar to that of *PUCHI* (Figure 1A; Supplementary Figure S5A).

**Figure 6:**
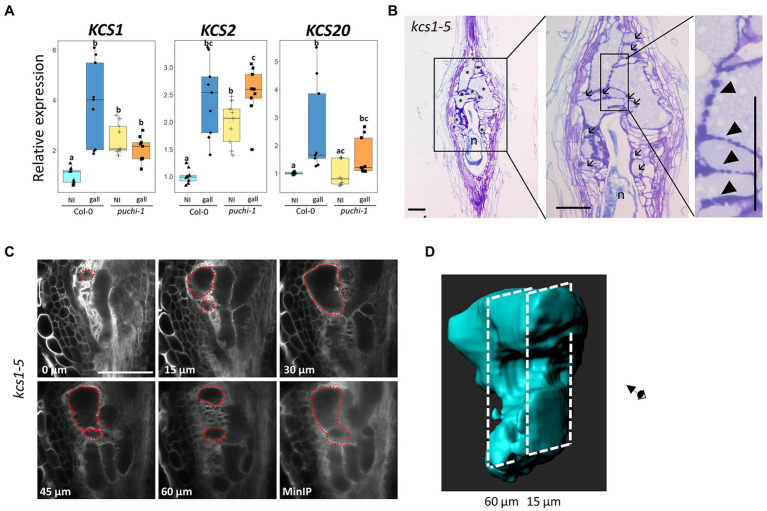
Expression analysis of VLCFA biosynthesis genes after RKN infection and morphological analysis of *kcs1-5* galls. **(A)** qRT-PCR results of *KCS1, KCS2*, and *KCS20* expression in WT (blue) and *puchi-1* (orange) with uninfected roots (brighter shade) and 5 dpi galls (darker shade). Values are means of three technical replicates. Three biological replicates were performed with similar results. Alphabets denote significant differences between groups (Steel-Dwass’s multiple comparisons test, p<0.05). **(B)** Toluidine blue-stained longitudinal sections of *kcs1-5* galls at 14 dpi. Arrows denote aberrant GCs, and arrowheads denote thickened lobate GC cell wall. ^*^ denotes GCs; n denotes RKNs. Scale bars: 100μm. **(C)** Confocal optical sections of *kcs1-5* galls stained with Calcofluor White at 7 dpi, and red dashed line outlines the same GC in different sections and the MinIP of the *kcs1-5* optical section Z-stacks. Scale bars: 100μm. **(D)** 3D reconstruction of individual *kcs1-5* GCs using the optical sections in **(C)**.

qRT-PCR results were consistent with the notion that RKN infection induces *KCS1* expression, since *KCS1* transcript levels did increase significantly upon RKN infection in WT plants. However, such was not the case in *puchi-1* plants, suggesting *PUCHI* positively regulates *KCS1* expression during RKN infection (Figure 6A). The expression level of *KCS20* also increased significantly after RKN infection in a *PUCHI*-dependent fashion; however, the magnitude of increase was much lower compared to *KCS1* (Figure 6A). In contrast, *KCS2* expression was significantly increased in galls of both WT and *puchi-1*, suggesting *KCS2* is unlikely to be regulated by *PUCHI* (Figure 6A). Expression levels of *BETA-KETOACYL REDUCTASE* 1 (*KCR1*), *PAS2*, and *ECERIFERUM* (*ECR*) did not show significant increase in neither WT nor *puchi-1* after RKN infection (Supplementary Figure S5B). These results suggested that among the VLCFA biosynthesis genes tested, only *KCS1* and *KCS20* were induced by *PUCHI* during gall formation. Since *KCS1* showed the most pronounced upregulation, we have decided to focus on *KCS1* for the subsequent analyses. To determine the converse of whether *KCS1* influences *PUCHI* expression, *PUCHI* expression was examined *kcs1-5* plants during RKN infection. No significant difference in *PUCHI* expression levels between *kcs1-5* and WT in gall was observed; however, *PUCHI* expression was slightly, albeit significantly, increased in uninfected roots of *kcs1-5* compared to the WT (Supplementary Figure S5C). This suggests that *PUCHI* may be transcriptionally repressed by events downstream of *KCS1* as a form of negative feedback.

GUS staining in *pKCS1::GUS* transgenic plants was observed primarily in both uninfected main roots, LRPs and RKN-induced galls (Supplementary Figure S5D). Moreover, the *puchi-1* mutation also caused a clear and consistent reduction of GUS staining in developing LRPs of *pKCS1::GUS* transgenic plants (Supplementary Figure S5D). In some 7 dpi *puchi pKCS1::GUS* galls, GUS staining was reduced to be restricted in the vascular region, whereas in the WT background, GUS staining can be found throughout the entire gall, consistent with the qRT-PCR results (Supplementary Figure S5D). Interestingly, GUS signal from *pKCS1::GUS* appears to be slightly stronger in the LRs of *puchi-1* plants compared to the WT (Supplementary Figure S5D). This may explain the slightly increased *KCS1* expression level in uninfected *puchi-1* plants compared to the WT (Figure 6A). Nevertheless, the GUS-reporter line analysis validates the notion that *KCS1* is positively regulated by *PUCHI* during gall formation.

To further investigate the roles of *KCS1* during GC development, *kcs1* GC morphology was observed. Accordingly, *kcs1-5* galls also exhibited aberrant GCs (Figure 6B; Supplementary Figures S5E,F) and thicker lobate cell walls (Figure 6B; Supplementary Figures S5E,G), similar to those observed in *puchi-1* galls, implying that *KCS1* may function in the same pathway as *PUCHI* in GC development. To confirm whether the aberrant GCs found in the *puchi* and *kcs1-5* galls were comparable, GC distributions (based on size) were also classified in 14 dpi WT and *kcs1-5* galls. Similar to the *puchi-1* gall findings, the *kcs1-5* galls showed an increase in the proportion of GCs with a cross-section area of 0–3,000μm^2^, and a decrease in the proportion of GCs with an area of 3,001–7,000μm^2^ (Figure 3E). The fact that both *puchi-1* and *kcs1-5* galls showed a shift toward smaller GCs in cross-sections may imply that both genes utilize the same mechanism to control GC morphology. Moreover, the 3D reconstruction of *kcs1-5* GCs also showed protrusions and invaginations on the surface, similar to *puchi-1* GCs (Figures 6C,D). Additionally, the WT, *puchi-1*, and *kcs1-5* galls all contained comparable proportions of GCs with a cross-section area above 7,000μm^2^ (Figure 3E), which is consistent with the finding that the overall GC size was comparable between the WT and *puchi* galls. The fact that *kcs1-5* phenocopies the *puchi-1* GC defects suggests that *KCS1* and *PUCHI* may function in the same pathway to regulate GC development.

## Discussion

### *PUCHI* Is Expressed During the Early Stages of Gall Formation

The RKN infestation process can be separated into three distinct stages: invasion (~1 dpi), GC induction (~4 dpi), and nutrient acquisition (~7 dpi; Bartlem et al., 2014). GC growth occurs during the nutrient acquisition stage (Jones and Northcote, 1972; Jones and Payne, 1978). In this study, we demonstrated that the AP2/EREBP-type transcription factor *PUCHI* is expressed during the early stages (2~7 dpi) of gall formation (Figures 1A,B). Observation of *ProPUCHI-GUS-tPUCHI* roots showed that *PUCHI* was not detected in any root tissues except the LRP of uninfected plants. However, following RKN infection, *PUCHI* expression was detected in the developing galls (Figure 1B). These results suggest that *PUCHI* may function during gall formation. qRT-PCR results confirmed that *PUCHI* was most highly expressed in galls at 5 dpi (Figure 1A). Therefore, these results indicate that *PUCHI* is likely to be involved in early stage GC development. However, we found that the number of invading RKNs, galls formed, mature females that emerged, and egg masses produced, and the gall sizes were not significantly different between *puchi* and WT galls (Figure 2; Supplementary Figure S2). It has been reported that the *Arabidopsis* AP2 transcription factors *PUCHI*, *DORNRÖSCHEN* (*DRN*), and *DORNRÖSCHEN-LIKE* (*DRNL*) redundantly control floral organ initiation within the floral meristem (Chandler and Werr, 2017). Therefore, it is possible that other genes may be functionally redundant with *PUCHI* in RKN development regulation, such that the *puchi* single mutant alone does not produce dramatic defects in RKN infectivity and development. In the future, we consider it necessary to analyze higher-order mutants with these paralogs.

### *PUCHI* Is Required for Proper GC Formation

In gall thin sections, the GCs of the *puchi* mutants were found to be smaller than those of the WT (Figure 3). However, 3D reconstructions of GCs generated using confocal microscopy optical sections, the aberrant *puchi* GCs were shown to be protrusions of larger GCs (Figure 5B; Supplementary Figure S4B). It seems that *puchi* galls form GCs that are more misshapen than those of WT galls and that the actual number of GCs does not change significantly. It is possible that steric constraints, such as the presence of nematodes, the rapid growth of GCs, and the presence of neighboring cells, result in the GC protrusions and indentations in WT plants (Cabrera et al., 2015, 2018). In *puchi-1*, these steric constraints may be more intense, as it has been reported that *PUCHI* represses cell divisions in LRPs (Hirota et al., 2007). Moreover, parts of the *puchi-1* GC cell wall appeared to be thicker and lobate when compared to the WT GC cell wall, and sometimes formed invaginations (Figures 3A–D; Supplementary Figure S3C). Therefore, another possible explanation of how *puchi-1* GCs fail to maintain their proper shape may be excessive cell membrane and cell wall material production, and we have indeed demonstrated that PUCHI may regulate VLCFA biosynthesis. Interestingly, the *pas2* mutant also shows cell wall defects that are visually similar to those seen in *puchi* galls in the absence of RKN infection, suggesting there may be RKN-independent factors leading to these cell wall defects (Bach et al., 2011). *PUCHI* has been shown to control cell proliferation during LRP formation, as the *puchi-1* mutant exhibits excessive cell division and produces abnormally enlarged flank cells in the early phase of LRP development (Hirota et al., 2007). Thus, it is possible that *PUCHI* not only suppresses mitosis in LRP formation, but also regulates cell wall formation in GCs.

### *PUCHI* May Control the Expression of VLCFA Biosynthesis Genes During Gall Formation

Cell walls between adjacent GCs were irregularly shaped in *puchi-1* galls, with some regions containing thicker walls with more elaborate lobes than found in WT galls (Figures 3A–D). This is a superficially similar result to the abortive cell plates observed in *pas2-1* mutants (Bach et al., 2011). In addition, it has been reported that *PUCHI* regulates the VLCFA biosynthesis pathway during lateral root development (Trinh et al., 2019). These lines of evidence imply that there may be a connection between gall formation and VLCFA biosynthesis *via* PUCHI. We have demonstrated that *KCS1* is indeed positively regulated by *PUCHI* during gall formation (Figure 6A), and the GCs of *kcs1-5* galls also showed thicker and lobate cell walls like those of *puchi-1* GCs. Furthermore, the aberrant GCs observed in *kcs1-5* galls were also found to be parts of larger GCs like in *puchi-1* galls (Figure 6D). This suggests that the GC defects seen in *puchi* mutants may be consequences of dysregulated VLCFA biosynthesis mediated by *KCS1*.

It should also be noted that a recent study showed that VLCFAs may play a role in the regulation of cellulose synthesis. The *pas2* mutant exhibits reduced cell elongation in dark-grown hypocotyls (Zhu et al., 2020). This defect is likely due to reductions in the cellulose detected in the primary cell wall, cellulose synthase complex secretion/motility in the plasma membrane (Zhu et al., 2020). It has been reported that cellulose-deficient mutants and plants treated with the cellulose synthesis inhibitor isoxaben showed abnormal cell shapes, such as swelling and reduction of elongation in hypocotyls and roots (Arioli et al., 1998; Fagard et al., 2000; Refrégier et al., 2004; Fujita et al., 2013). These lines of evidence suggest a cross-regulatory network between VLCFAs, cell wall composition, and cell morphology. In short, the composition of the *puchi-1* cell wall may have changed due to alterations in the cell membrane VLCFA composition. Both the cell wall carbohydrates and the cell membrane VLCFAs of the *puchi-1* mutant may contribute to cell shape deformities, as either the altered cell wall and/or cell membrane may have buckled under the pressure exerted by the various steric hindrances in galls as described above.

## Conclusion

Our findings suggest that PUCHI may be involved in the development of GCs *via* the regulation of VLCFA biosynthesis. Subsequent research will hopefully identify the pathway responsible for these processes, provide knowledge about how nematodes modulate GC and neighboring cell development, and further our understanding of PUCHI’s role in plant development.

## Data Availability Statement

The datasets presented in this study can be found in online repositories. The names of the repository/repositories and accession number(s) can be found at: DDBJ [accession: PRJDB5797].

## Author Contributions

RS, TH, MA, and MK conceived and designed the experiments. RS, MY, and TH performed the experiments. RS, AT, and SS wrote the manuscript. MK, TH, MA, AT, and SS revised the manuscript. All authors contributed to the article and approved the submitted version.

## Funding

This research was funded by the JSPS KAKENHI (grant numbers: 18H05487, 20H00422, 20KK0135, and 21K19273 to SS).

## Conflict of Interest

The authors declare that the research was conducted in the absence of any commercial or financial relationships that could be construed as a potential conflict of interest.

## Publisher’s Note

All claims expressed in this article are solely those of the authors and do not necessarily represent those of their affiliated organizations, or those of the publisher, the editors and the reviewers. Any product that may be evaluated in this article, or claim that may be made by its manufacturer, is not guaranteed or endorsed by the publisher.

## Abbreviations

GCs: Giant Cells
RKNs: Root-Knot Nematodes
RNA-Seq: RNA Sequencing
LR: Lateral Root
MinIP: Minimum Intensity Projection
VLCFA: Very Long Chain Fatty Acid

## References

[ref1] Arioli T. Peng L. Betzner A. S. Burn J. Wittke W. Herth W.. (1998). Molecular analysis of cellulose biosynthesis in *Arabidopsis*. Science 279, 717–720. doi: 10.1126/science.279.5351.717, PMID: 9445479

[ref2] Bach L. Gissot L. Marion J. Tellier F. Moreau P. Satiat-Jeunemaître B.. (2011). Very-long-chain fatty acids are required for cell plate formation during cytokinesis in *Arabidopsis thaliana*. J. Cell Sci. 124, 3223–3234. doi: 10.1242/jcs.074575, PMID: 21896643

[ref3] Bartlem D. G. Jones M. G. K. Hammes U. Z. (2014). Vascularization and nutrient delivery at root-knot nematode feeding sites in host roots. J. Exp. Bot. 65, 1789–1798. doi: 10.1093/jxb/ert415, PMID: 24336493

[ref4] Bird A. F. (1961). The ultrastructure and histochemistry of a nematode-induced giant-cell. J. Biophys. Biochem. Cytol. 11, 701–715. doi: 10.1083/jcb.11.3.701, PMID: 13869341 PMC2225126

[ref5] Cabrera J. Díaz-Manzano F. E. Barcala M. Arganda-Carreras I. Almeida-Engler J. de Engler G.. (2015). Phenotyping nematode feeding sites: three-dimensional reconstruction and volumetric measurements of giant cells induced by root-knot nematodes in *Arabidopsis*. New Phytol. 206, 868–880. doi: 10.1111/nph.13249, PMID: 25613856

[ref6] Cabrera J. Díaz-Manzano F. E. Sanchez M. Rosso M.-N. Melillo T. Goh T.. (2014). A role for LATERAL ORGAN BOUNDARIES-DOMAIN 16 during the interaction *Arabidopsis*–*Meloidogyne* spp. provides a molecular link between lateral root and root-knot nematode feeding site development. New Phytol. 203, 632–645. doi: 10.1111/nph.12826, PMID: 24803293

[ref7] Cabrera J. Olmo R. Ruiz-Ferrer V. Abreu I. Hermans C. Martinez-Argudo I.. (2018). A phenotyping method of giant cells from root-knot nematode feeding sites by confocal microscopy highlights a role for CHITINASE-LIKE 1 in *Arabidopsis*. Int. J. Mol. Sci. 19:429. doi: 10.3390/ijms19020429, PMID: 29389847 PMC5855651

[ref8] Caillaud M.-C. Dubreuil G. Quentin M. Perfus-Barbeoch L. Lecomte P. de Almeida Engler J.. (2008). Root-knot nematodes manipulate plant cell functions during a compatible interaction. J. Plant Physiol. 165, 104–113. doi: 10.1016/j.jplph.2007.05.007, PMID: 17681399

[ref9] Chandler J. Werr W. (2017). DORNRÖSCHEN, DORNRÖSCHEN-LIKE, and PUCHI redundantly control floral meristem identity and organ initiation in *Arabidopsis*. J. Exp. Bot. 68, 3457–3472. doi: 10.1093/jxb/erx208, PMID: 28859377

[ref10] Fagard M. Desnos T. Desprez T. Goubet F. Refregier G. Mouille G.. (2000). PROCUSTE1 encodes a cellulose synthase required for normal cell elongation specifically in roots and dark-grown hypocotyls of *Arabidopsis*. Plant Cell 12, 2409–2423. doi: 10.1105/tpc.12.12.2409, PMID: 11148287 PMC102227

[ref11] Favery B. Quentin M. Jaubert-Possamai S. Abad P. (2016). Gall-forming root-knot nematodes hijack key plant cellular functions to induce multinucleate and hypertrophied feeding cells. J. Insect Physiol. 84, 60–69. doi: 10.1016/j.jinsphys.2015.07.013, PMID: 26211599

[ref12] Fujita M. Himmelspach R. Ward J. Whittington A. Hasenbein N. Liu C.. (2013). The anisotropy1 D604N mutation in the *Arabidopsis* cellulose synthase1 catalytic domain reduces cell wall crystallinity and the velocity of cellulose synthase complexes. Plant Physiol. 162, 74–85. doi: 10.1104/pp.112.211565, PMID: 23532584 PMC3641231

[ref13] Goh T. Toyokura K. Yamaguchi N. Okamoto Y. Uehara T. Kaneko S.. (2019). Lateral root initiation requires the sequential induction of transcription factors LBD16 and PUCHI in *Arabidopsis thaliana*. New Phytol. 224, 749–760. doi: 10.1111/nph.16065, PMID: 31310684

[ref14] Guyomarc’h S. Boutté Y. Laplaze L. (2021). AP2/ERF transcription factors orchestrate very long chain fatty acid biosynthesis during *Arabidopsis* lateral root development. Mol. Plant 14, 205–207. doi: 10.1016/j.molp.2021.01.004, PMID: 33450371

[ref15] Henikoff S. Till B. J. Comai L. (2004). TILLING. Traditional mutagenesis meets functional genomics. Plant Physiol. 135, 630–636. doi: 10.1104/pp.104.041061, PMID: 15155876 PMC514099

[ref16] Hirota A. Kato T. Fukaki H. Aida M. Tasaka M. (2007). The auxin-regulated AP2/EREBP gene PUCHI is required for morphogenesis in the early lateral root primordium of *Arabidopsis*. Plant Cell 19, 2156–2168. doi: 10.1105/tpc.107.050674, PMID: 17630277 PMC1955702

[ref17] Jones M. G. K. Northcote D. H. (1972). Multinucleate transfer cells induced in coleus roots by the root-knot nematode, *Meloidogyne arenaria*. Protoplasma 75, 381–395. doi: 10.1007/BF01282117

[ref18] Jones M. G. K. Payne H. L. (1978). Early stages of nematode-induced giant-cell formation in roots of *Impatiens balsamina*. J. Nematol. 10, 70–84. PMID: 19305816 PMC2617858

[ref19] Joubès J. Raffaele S. Bourdenx B. Garcia C. Laroche-Traineau J. Moreau P.. (2008). The VLCFA elongase gene family in Arabidopsis thaliana: phylogenetic analysis, 3D modelling and expression profiling. Plant Mol. Biol. 67:547. doi: 10.1007/s11103-008-9339-z, PMID: 18465198

[ref20] Karim M. R. Hirota A. Kwiatkowska D. Tasaka M. Aida M. (2009). A role for *Arabidopsis PUCHI* in floral meristem identity and bract suppression. Plant Cell 21, 1360–1372. doi: 10.1105/tpc.109.067025, PMID: 19482972 PMC2700531

[ref21] Karnowski K. Ajduk A. Wieloch B. Tamborski S. Krawiec K. Wojtkowski M.. (2017). Optical coherence microscopy as a novel, non-invasive method for the 4D live imaging of early mammalian embryos. Sci. Rep. 7:4165. doi: 10.1038/s41598-017-04220-8 28646146 PMC5482811

[ref22] Kurihara D. Mizuta Y. Sato Y. Higashiyama T. (2015). ClearSee: a rapid optical clearing reagent for whole-plant fluorescence imaging. Development 142, 4168–4179. doi: 10.1242/dev.127613, PMID: 26493404 PMC4712841

[ref23] Li-Beisson Y. Shorrosh B. Beisson F. Andersson M. X. Arondel V. Bates P. D.. (2013). Acyl-lipid metabolism. Arabidopsis Book 11:e0161. doi: 10.1199/tab.0161, PMID: 23505340 PMC3563272

[ref24] Nishiyama H. Ngan B. T. Nakagami S. Ejima C. Ishida T. Sawa S. (2015). Protocol for root-knot nematode culture by a hydroponic system and nematode inoculation to *Arabidopsis*. Jpn. J. Nematol. 45, 45–49. doi: 10.3725/jjn.45.45

[ref25] Olmo R. Cabrera J. Díaz-Manzano F. E. Ruiz-Ferrer V. Barcala M. Ishida T.. (2020). Root-knot nematodes induce gall formation by recruiting developmental pathways of post-embryonic organogenesis and regeneration to promote transient pluripotency. New Phytol. 227, 200–215. doi: 10.1111/nph.16521, PMID: 32129890

[ref26] Olmo R. Cabrera J. Moreno-Risueno M. A. Fukaki H. Fenoll C. Escobar C. (2017). Molecular transducers from roots are triggered in *Arabidopsis* leaves by root-knot nematodes for successful feeding site formation: a conserved post-embryogenic de novo organogenesis program? Front. Plant Sci. 8:875. doi: 10.3389/fpls.2017.00875 28603536 PMC5445185

[ref27] Refrégier G. Pelletier S. Jaillard D. Höfte H. (2004). Interaction between wall deposition and cell elongation in dark-grown hypocotyl cells in *Arabidopsis*. Plant Physiol. 135, 959–968. doi: 10.1104/pp.104.038711, PMID: 15181211 PMC514130

[ref28] Rodiuc N. Vieira P. Banora M. Y. de Almeida Engler J. (2014). On the track of transfer cell formation by specialized plant-parasitic nematodes. Front. Plant Sci. 5:160. doi: 10.3389/fpls.2014.00160, PMID: 24847336 PMC4017147

[ref29] Shang B. Xu C. Zhang X. Cao H. Xin W. Hu Y. (2016). Very-long-chain fatty acids restrict regeneration capacity by confining pericycle competence for callus formation in *Arabidopsis*. Proc. Natl. Acad. Sci. U. S. A. 113, 5101–5106. doi: 10.1073/pnas.1522466113, PMID: 27092001 PMC4983810

[ref30] Takano S. Niihama M. Smith H. M. S. Tasaka M. Aida M. (2010). *Gorgon*, a novel missense mutation in the *SHOOT MERISTEMLESS* gene, impairs shoot meristem homeostasis in *Arabidopsis*. Plant Cell Physiol. 51, 621–634. doi: 10.1093/pcp/pcq028, PMID: 20208065

[ref31] Trinh D.-C. Lavenus J. Goh T. Boutté Y. Drogue Q. Vaissayre V.. (2019). PUCHI regulates very long chain fatty acid biosynthesis during lateral root and callus formation. Proc. Natl. Acad. Sci. U. S. A. 116, 14325–14330. doi: 10.1073/pnas.1906300116, PMID: 31235573 PMC6628829

[ref32] Wiggers R. J. Starr J. L. Price H. J. (1990). DNA content and variation in chromosome number in plant cells affected by *Meloidogyne incognita* and *M. arenaria*. Phytopathology 80, 1391–1395. doi: 10.1094/Phyto-80-1391

[ref33] Williamson V. M. Gleason C. A. (2003). Plant–nematode interactions. Curr. Opin. Plant Biol. 6, 327–333. doi: 10.1016/S1369-5266(03)00059-1 12873526

[ref34] Yamaguchi Y. L. Suzuki R. Cabrera J. Nakagami S. Sagara T. Ejima C.. (2017). Root-knot and cyst nematodes activate procambium-associated genes in *Arabidopsis* roots. Front. Plant Sci. 8:1995. doi: 10.3389/fpls.2017.01195 28747918 PMC5506325

[ref35] Zhu X. Tellier F. Gu Y. Li S. (2020). Disruption of very-long-chain-fatty acid synthesis has an impact on the dynamics of cellulose synthase in *Arabidopsis thaliana*. Plan. Theory 9:1599. doi: 10.3390/plants9111599, PMID: 33218005 PMC7698757

