# Supplementary material for: PUCHI Regulates Giant Cell Morphology During Root-Knot Nematode Infection in Arabidopsis thaliana
Source: Front Plant Sci. 2021 Oct 6;12:755610. doi: 10.3389/fpls.2021.755610 (PMC8527015; doi:10.3389/fpls.2021.755610)
Supplement: Supplementary file 1 [file Data_Sheet_1.PDF]

Supplementary Table S1. **List of primer sequences used for qRT-PCR analysis**

Gene name	Primer name	Primer sequence (5' - 3')
GAPDH	GAPDHqRT-PCR-F1	TTAGTCGCAACCTGAAGCCATC
	GAPDHqRT-PCR-R1	TTCCACTGCTACTTGACCTTCG
PUCHI	PUCHI-LCL	ACGGCTCGTTATCTTCTTCACT
	PUCHI-LCR	TGGACTTATTATGTTCTTCGCTTG
KCS1	KCS1 F	CTTGCAACGTGACCACCAT
	KCS1 R	AGCACGGTTCCGGTTAAAG
KCS2	KCS2 F	CCATTGATCTCGCTAAACAGC
	KCS2 R	TCGGTCGTTGCCTAAATACC
KCS20	KCS20 F	GCTTAGAGGCAACATTTTGAGC
	KCS20 R	GCGTATGAGTTTGGTTGCAC
KCR1	KCR1 F	GCTTAAGAGGAAGAAAGGTGCTATT
	KCR1 R	CACTTTGTGAACTGATCCACGTA
PAS2	PAS2 F	TCTATGACGCCATTGAGAAGC
	PAS2 R	CAGGAGATCTGACCAAACCTACTAA
ECR	ECR F	CCTTGACCTCCCCGATTC
	ECR R	CCAGGAGTCACGGGAAGA

SFig1

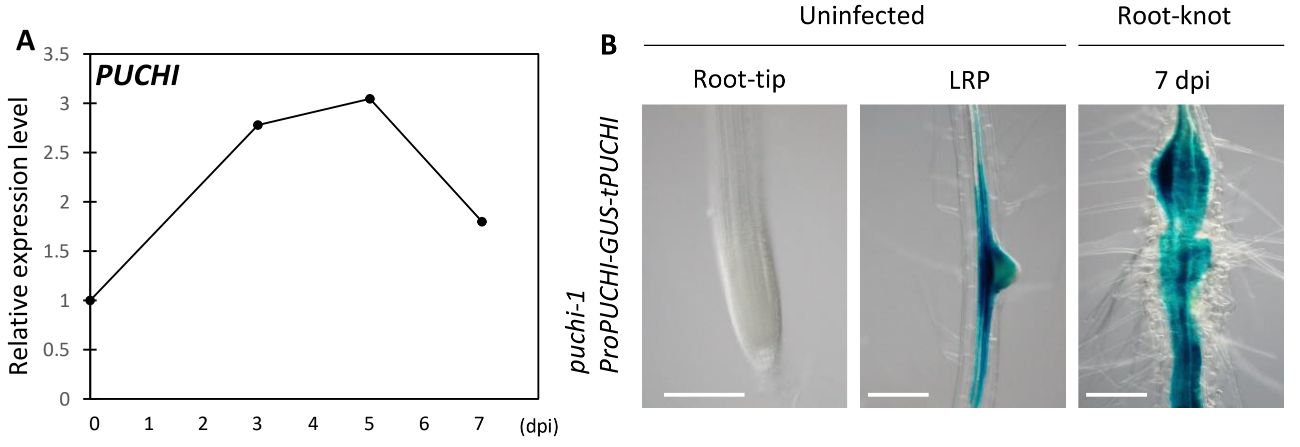

Figure S1. **Expression analysis of *PUCHI* after RKN infection and in *puchi-1* galls.** (A) *PUCHI* mRNA levels in developing galls. Values are the average of two experiments normalized to the mRNA level before infection, adapted from Yamaguchi et al. (2017). (B) GUS-stained *puchi-1*, *ProPUCHI-GUS-tPUCHI* 7 dpi galls, and uninfected roots, to denote the spatial expression patterns of *PUCHI* in the absence of the genomic *PUCHI* locus. Scale bars: 100 µm.

SFig2

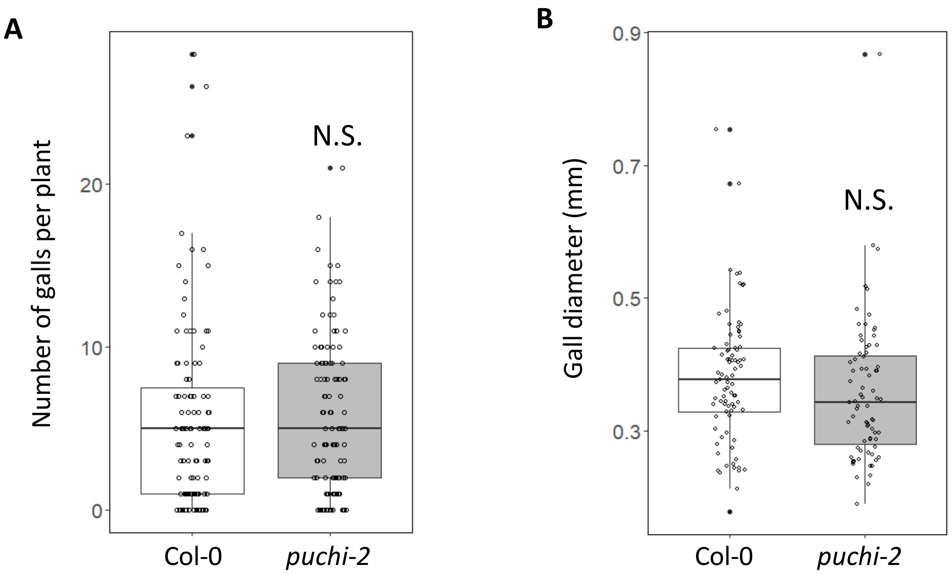

Figure S2. **Effects of *puchi-2* mutation during RKN infection.** Box plots of the number of galls (A) and gall diameters at 14 dpi (B) in the Col-0 and *puchi-2* samples. Values are the means of at least three biological replicates. Statistical significance was analyzed using the Brunner-Munzel test. N.S. denotes not statistically significant.

FigS3

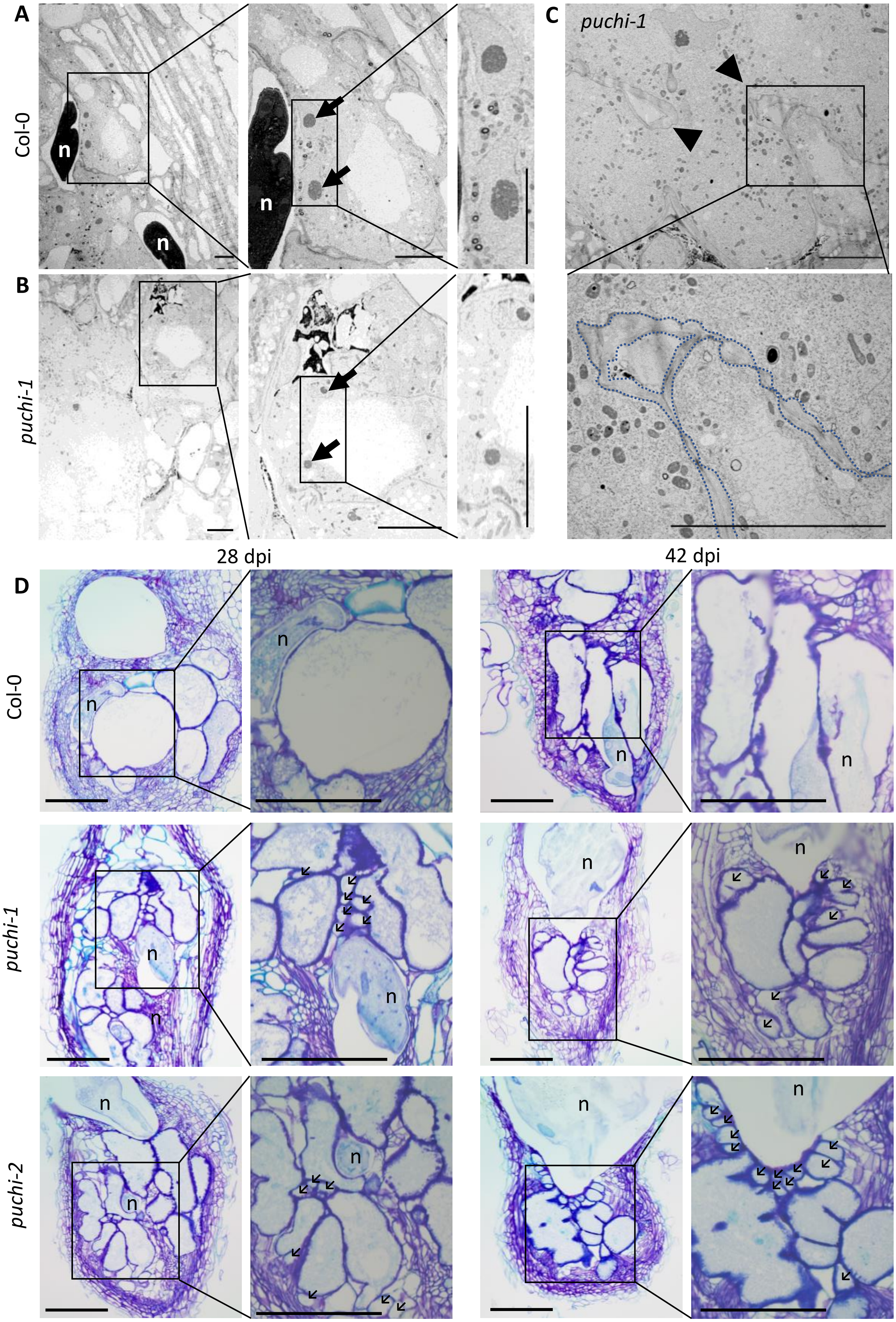

Figure S3. **Observation of the GC morphology of WT and *puchi-1* and *puchi-2* mutants.** (A, B) TEM micrographs of WT and *puchi-1* gall sections, arrowheads denote nuclei in WT (A) and *puchi-1* (B) galls, scale bar: 10 µm. (C) TEM micrographs of *puchi-1* gall sections, arrowhead denotes invaginated cell wall and blue dotted lines outline cell boundaries, n denotes RKNs, scale bar: 10 µm. (D) Toluidine blue-stained longitudinal sections of WT, *puchi-1*, and *puchi-2* galls at 28 and 42 dpi. Arrows denote aberrant GCs, n denotes RKNs, scale bar: 100 µm.

FigS4

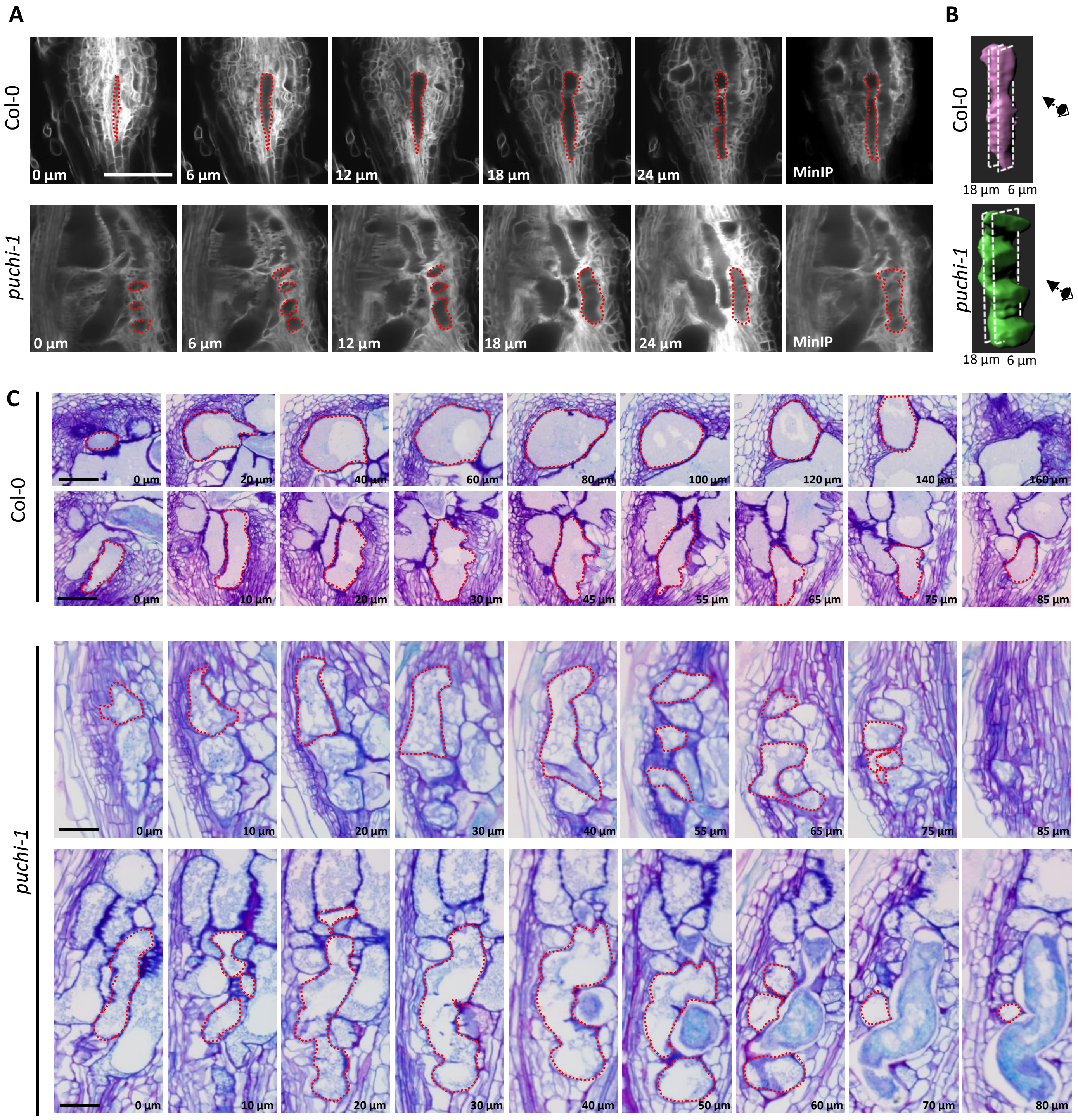

Figure S4. **3D structures of the WT and *puchi-1* GCs.** (A) Confocal optical sections of additional WT and *puchi-1* galls stained with Calcofluor White at 7 dpi, Scale bars: 100 µm. (B) 3D reconstruction of individual WT and *puchi-1* GCs using the optical sections in (A). (C) Toluidine blue-stained longitudinal sections of WT and *puchi-1* galls at 14 dpi, red dashed lines outline the same GCs in different optical sections. Scale bars: 50 µm. Red dashed lines indicate the same GC confirmed by 3D reconstruction.

FigS5

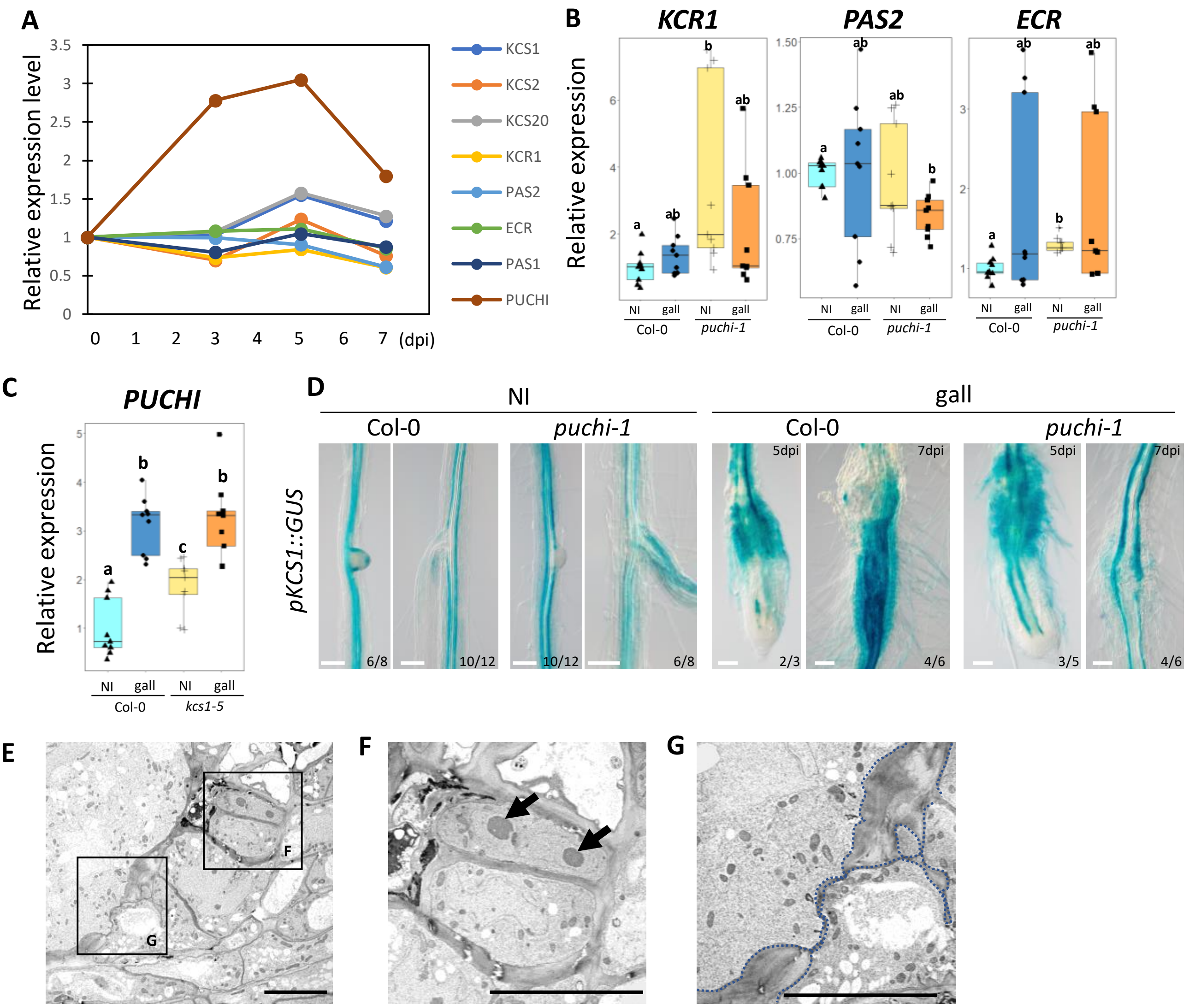

Figure S5. **Expression analysis of VLCFA biosynthesis genes after RKN infection and morphological analysis of *kcs1-5* galls.** (A) Relative expression levels of VLCFA biosynthesis genes *KCS1*, *KCS2*, *KCR1*, *PAS2*, *ECR*, and *PAS1* during WT gall development based on RNA-Seq data from Yamaguchi et al., 2017, values are the average of two biological replicates normalized to the expression level before infection. (B) qRT-PCR results of *KCR1*, *PAS2*, and *ECR* expression in WT (blue) and *puchi-1* (orange) with uninfected roots (brighter shade) and 5 dpi galls (darker shade). Values are means of three technical replicates. Three biological replicates were performed with similar results. Alphabets denote significant differences between groups (Steel-Dwass's multiple comparisons test, P<0.05). (C) qRT-PCR results of *PUCHI* expression level in WT (blue) and *kcs1-5* (orange) with uninfected roots (brighter shade) and 5 dpi galls (darker shade). Values are means of three technical replicates. Three biological replicates were performed with similar results. Alphabets denote significant differences between groups (Steel-Dwass's multiple comparisons test, P<0.05). (D) GUS-stained 5 and 7 dpi galls and uninfected roots of *pKCS1::GUS* transgenic plants in the Col-0 and *puchi-1* backgrounds. Scale bars: 100 µm. (E) TEM micrographs of *kcs1-5* gall sections at 7 dpi, (F) a close-up view of aberrant GCs in (E), arrowheads denote nuclei, and (G) a close-up view of the GC boundary in (E). Blue dotted lines denote invaginated cell boundaries. Scale bars: 10 µm.
